# Engineering strategies for cartilage endplate reconstruction: a tissue engineering perspective

**DOI:** 10.1097/JS9.0000000000005125

**Published:** 2026-03-20

**Authors:** Yifan Wang, Chuyue Zhang, Zheng Wang

**Affiliations:** Senior Department of Orthopedics, The Fourth Medical Center of Chinese PLA General Hospital, Beijing, China

The intervertebral disc is a fibrocartilaginous structure connecting adjacent vertebrae, serving as the primary mechanical shock-absorbing core of the spinal column. Its structural integrity is essential for maintaining spinal stability, flexibility, and load-bearing capacity. A fully functional intervertebral disc consists of three distinct anatomical components: the centrally located nucleus pulposus, the peripherally arranged annulus fibrosus, and the superior and inferior cartilaginous endplates[1]. The cartilaginous endplate, which forms the critical interface between the bony vertebral body and the soft tissue of the disc, holds particular anatomical and functional significance. A normal cartilaginous endplate comprises a thin layer of hyaline cartilage, approximately 0.6–1 mm in thickness, devoid of vascular and neural elements but permeated with microscopic pores. It functions not only as a mechanical interface that distributes spinal loads but also serves as the principal conduit for the entire intervertebral disc, which is the largest avascular structure in the human body. Over 75% of the nutritional supply, including essential nutrients such as glucose and oxygen, along with the removal of metabolic waste products, depends on the passive diffusion capacity of the cartilaginous endplate. This physiological process is directly governed by the appropriate porosity and permeability maintained by its extracellular matrix composition, predominantly type II collagen and proteoglycans^[^2,3^]^.

However, degeneration of the cartilaginous endplate frequently represents the initial triggering event and a central driving mechanism in the pathological process of intervertebral disc degeneration. Multiple factors, including aging, abnormal mechanical loading, genetic predisposition, and inflammatory responses, can initiate pathological remodeling of the cartilaginous endplate. Characteristic pathological alterations include cellular senescence, apoptosis, and ferroptosis of cartilaginous endplate cells; an imbalance in extracellular matrix metabolism manifested by reduced synthesis and accelerated degradation leading to the substantial loss of type II collagen and proteoglycans; and most critically, pathological calcification. These pathological changes collectively result in thickening, hardening, and occlusion of the microporous structure of the cartilaginous endplate, consequently causing a significant decline in solute diffusion efficiency. The interruption of nutritional supply pathways coupled with the accumulation of metabolic waste products jointly contribute to nucleus pulposus cell dysfunction and accelerated extracellular matrix breakdown, ultimately establishing an irreversible degenerative vicious cycle^[^4,5^]^. Furthermore, degenerated cartilaginous endplates release substantial quantities of pro-inflammatory cytokines such as IL-1β and TNF-α, and exhibit abnormal vascular and neural infiltration, which not only exacerbates the local inflammatory microenvironment but also constitutes a significant etiology of chronic low back pain[6].

Based on these anatomical and pathophysiological characteristics, the design of biomaterials targeted at cartilaginous endplate repair must adhere to several fundamental principles. The primary objective is the reestablishment of nutritional transport pathways. Calcification and chondrocyte apoptosis within the cartilaginous endplate obstruct the disc’s sole nutritional portal. Ma *et al*[7] demonstrated that CAP-Nrf2-exosomes specifically target cartilaginous endplate chondrocytes and inhibit Drp1-mediated mitochondrial fission and apoptosis, thereby preserving cellular density and architectural integrity of the endplate. Concurrent activation of the Nrf2 antioxidant signaling cascade protects residual microvascular structures, ensuring sustained diffusion of glucose, oxygen, and metabolites into the nucleus pulposus region.

A crucial strategic approach involves the precise regulation of cartilaginous endplate cell fate. Reprogramming the differentiation pathways of endplate stem cells or chondrocytes can fundamentally impede the degeneration process. Wang *et al*[8] developed an injectable extracellular matrix gel loaded with Sphk2-overexpressing cartilaginous endplate stem cells. These cells continuously release exosomes that activate the PI3K/Akt-autophagy signaling axis in nucleus pulposus cells, effectively inhibiting cellular senescence and promoting extracellular matrix synthesis. Cheng *et al*[9] further engineered CAP-Lipo@CEBPE nanoparticles that reconstruct the CEBPE-LTF-STAT3 regulatory circuit within chondrocytes, significantly suppressing inflammatory responses and calcification processes while restoring a hyaline cartilage phenotype. Both technological platforms achieve integrated material, genetic, and cellular functionalities, offering innovative solutions for endplate regeneration.

Counteracting the pathological microenvironment remains equally critical. Effective neutralization of acidic substances, reactive oxygen species, and inflammatory mediators is essential for ensuring the survival of both cartilaginous endplate and nucleus pulposus cells. Zhu *et al*[10] reported a CaCO3/chitosan composite hydrogel system capable of neutralizing hydrogen ions generated from lactic acid metabolism, while simultaneously delivering salvianolic acid A via CAP-guided exosomes to inhibit the iron-cGAS-STING signaling pathway in both cartilaginous endplate and nucleus pulposus cells. The material’s unique acid-responsive release characteristics enable rapid local alkalinization, antioxidant, and anti-inflammatory effects, creating a permissive microenvironment conducive to resident cell survival and function.

In summary, future biomaterials for cartilaginous endplate regeneration will transcend the conventional role of passive physical scaffolds. Instead, they are evolving into integrated, intelligent therapeutic platforms that combine the multifunctional capabilities of permeability enhancers, cellular behavior modulators, and microenvironment stabilizers.

## Data Availability

The data presented in this study are available in this article.
